# Systematic review of interventions for the prevention and treatment of postoperative urinary retention

**DOI:** 10.1002/bjs5.50114

**Published:** 2018-11-19

**Authors:** J. Jackson, P. Davies, N. Leggett, M. D. Nugawela, L. J. Scott, V. Leach, A. Richards, A. Blacker, P. Abrams, J. Sharma, J. Donovan, P. Whiting

**Affiliations:** ^1^ National Institute for Health Research Collaboration for Leadership in Applied Health Research and Care West (NIHR CLAHRC West) University Hospitals Bristol NHS Foundation Trust Bristol UK; ^2^ Population Health Sciences, Bristol Medical School University of Bristol Bristol UK; ^3^ Bristol Urological Institute, North Bristol NHS Trust Bristol UK; ^4^ University Hospitals Coventry and Warwickshire Coventry UK

## Abstract

**Background:**

Postoperative urinary retention (PO‐UR) is an acute and painful inability to void after surgery that can lead to complications and delayed hospital discharge. Standard treatment with a urinary catheter is associated with a risk of infection and can be distressing, undignified and uncomfortable. This systematic review aimed to identify effective interventions for the prevention and treatment of PO‐UR that might be alternatives to urinary catheterization.

**Methods:**

Electronic databases were searched from inception to September 2017. Randomized trials of interventions for the prevention or treatment of PO‐UR were eligible for inclusion. Studies were assessed for risk of bias using the Cochrane (2.0) tool. Two reviewers were involved at all review stages. Where possible, data were pooled using random‐effects meta‐analysis. The overall quality of the body of evidence was rated using the GRADE approach.

**Results:**

Some 48 studies involving 5644 participants were included. Most interventions were pharmacological strategies to prevent PO‐UR. Based on GRADE, there was high‐certainty evidence to support replacing morphine in a regional anaesthetic regimen, using alpha‐blockers (number needed to treat to prevent one case of PO‐UR (NNT) 5, 95 per cent c.i. 5 to 7), the antispasmodic drug drotaverine (NNT 9, 7 to 30) and early postoperative mobilization (NNT 5, 4 to 8) for prevention, and employing hot packs or gauze soaked in warm water for treatment (NNT 2, 2 to 4). Very few studies reported on secondary outcomes of pain, incidence of urinary tract infection or duration of hospital stay.

**Conclusion:**

Promising interventions exist for PO‐UR, but they need to be evaluated in randomized trials investigating comparative clinical and cost effectiveness, and acceptability to patients.

## Introduction

Postoperative urinary retention (PO‐UR) is frequently regarded as a minor adverse side‐effect of surgery, easily resolved by catheterization. The condition can, however, be distressing. Patients may find catheterization invasive, undignified and uncomfortable. Catheterization also carries risks of urinary tract infection^1^, which may lead to further complications such as infection of a joint prosthesis^2^. Untreated, PO‐UR can lead to overdistension of the bladder with acute kidney injury and detrusor muscle damage^3^. These events can result in delayed hospital discharge^4^ and additional care following hospital discharge.

Multiple perioperative predictors have been proposed for the development of PO‐UR, including age, sex, type of anaesthesia and analgesia^3^ ^5^. The varying standards and definitions adopted by different studies, along with the multifactorial aetiology of PO‐UR, are reflected by the wide‐ranging reported incidence for this problem of between 5·7 and 69 per cent^6^. Surgical populations at high risk of developing PO‐UR include joint arthroplasty (10·7–84 per cent), anorectal surgery (1–52 per cent), hernia repair (5·9–38 per cent)^3^ and gynaecological surgery (4–15 per cent)^7^.

Interventions to prevent or treat PO‐UR include pharmacological (such as cholinergic agents, alpha‐adrenergic blockers), massage, acupuncture and warm compress approaches. Interventions targeting anaesthesia and analgesia represent a potential preventive strategy. A clinical protocol of interventions effective in preventing PO‐UR, and treatment alternatives to catheterization, could lead to improved postoperative morbidity, reduction in associated complications, and improvements for patients with respect to dignity, comfort and psychological wellbeing.

The aim of this systematic review was to provide an overall summary of the available trial evidence on interventions to prevent or treat urinary retention after surgery.

## Methods

This review followed Cochrane recommendations for conducting systematic reviews^8^. The objectives, eligibility criteria and review methods were prespecified in a protocol registered with PROSPERO (CRD42016048765)^9^.

### Study selection

RCTs investigating any intervention with the specific aim of preventing or treating PO‐UR in an adult surgical population were included. Where the intervention was aimed at preventing PO‐UR, any adult surgical population was included. Where an intervention was aimed at treating PO‐UR, any adult surgical population experiencing PO‐UR according to the authors' definition was included. Populations with known pre‐existing urinary problems, such as benign prostatic obstruction, cancer of the bladder or prostate, or urinary incontinence, were excluded. Studies had to report incidence of PO‐UR, defined as urinary retention with a postoperative onset requiring urgent intervention, such as catheter insertion, recatheterization (where catheter use was part of the treatment protocol), pharmacological or non‐pharmacological treatment. Catheter use was considered as a proxy measure of PO‐UR (if not stated explicitly that the catheter was inserted to treat urinary retention). Outcomes defined as ‘postoperative voiding dysfunction’, ‘voiding difficulties’ or based on a postvoid residual urinary bladder volume alone were not considered PO‐UR.

### Identification and selection of studies

The following electronic databases were searched from inception to September 2017: MEDLINE (OVID) including MEDLINE in process, Embase (OVID), Cochrane Library (Wiley), Cumulative Index to Nursing and Allied Health Literature (CINAHL) (EBSCO), Science Citation Index and Conference proceedings – Science (Web of Science). The search strategies were developed specifically for each database (MEDLINE example included in *Appendix S1*, supporting information). Searches were limited to studies in humans, but were not limited by language, date or publication status.

Supplementary internet searches were undertaken to identify grey literature, including ongoing and completed clinical trials from the WHO International Clinical Trials Registry (www.who.int/ictrp/en) and National Institutes of Health Clinical Trials (clinicaltrials.gov).

The review team included a public contributor with personal experience of PO‐UR and researchers from the National Institute for Health Research Collaboration for Leadership in Applied Health Research and Care West (NIHR CLAHRC West). Taking on the role of a reviewer, the public contributor was actively involved in all aspects of screening and data extraction. Titles and abstracts were each screened independently by two members of the review team. All references that did not meet the inclusion criteria were excluded. Full‐text papers for each of the remaining references were obtained and examined independently in detail by two reviewers. Discrepancies between reviewers at each stage were resolved by discussion with a third reviewer.

### Data extraction and study appraisal

Data were extracted using a standard form developed in Microsoft Access^®^ 2010 (Microsoft, Redmond, Washington, USA) to include: eligibility criteria, study characteristics, participant characteristics, intervention, comparator, outcome definitions and outcome data for incidence of PO‐UR. Data were also extracted for: rates of urinary tract infection (UTI), duration of hospital stay, patient acceptability, adverse events (for example mortality) and pain scores. Data extraction was performed by one reviewer and checked by a second; disagreements were resolved by discussion with a third reviewer. Where PO‐UR was reported at multiple time points, data for the initial time point were extracted.

Included studies were assessed independently for risk of bias by two reviewers, using the Cochrane Risk of Bias tool (ROB 2.0)^10^ ^11^. This includes assessment criteria covering selection bias (random sequence generation and allocation concealment), performance bias (participant blinding), detection bias (blinding of outcome assessors), attrition bias (incomplete outcome data) and reporting bias (selective reporting).

### Statistical analysis

Comparisons were grouped by aim (prevention, treatment), intervention type (pharmacological, non‐pharmacological) and intervention category. For dichotomous data, an odds ratio (OR) for each comparison with associated 95 per cent confidence intervals was calculated. Continuous data were analysed as the weighted mean difference (MD) between groups and presented with associated 95 per cent confidence intervals. Where there were two or more studies in an intervention category, data were pooled using random‐effects meta‐analysis. To avoid double‐counting, where multiple different interventions were compared with the same control group, the control group was split into two or more smaller groups of equal size before being entered into the analysis. This approach permitted retention of as many relevant comparisons as possible^8^. If the study compared multiple different doses of the same drug with a control group, intervention groups were combined and data were entered into the meta‐analysis as a single comparison. Heterogeneity within groups was investigated using visual inspection of forest plots and quantified using the *I* 2 statistic^12^ ^13^. Where it was considered inappropriate to pool data, a narrative synthesis is provided.

The GRADE approach was used to rate the overall quality of the body of evidence for risk of bias, publication bias, imprecision, inconsistency and indirectness. GRADE ratings of very low, low, moderate or high certainty measure the overall quality of the evidence and reflect confidence in the evidence for each effect estimate^14^.

## Results

**Figure 1:**
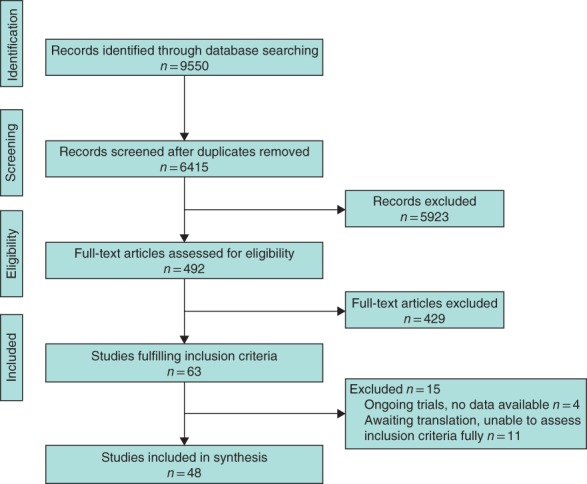
PRISMA flow diagram for the review

After removal of duplicate records, 6415 reports were identified of which 48 studies^15^, ^16^, ^17^, ^18^, ^19^, ^20^, ^21^, ^22^, ^23^, ^24^, ^25^, ^26^, ^27^, ^28^, ^29^, ^30^, ^31^, ^32^, ^33^, ^34^, ^35^, ^36^, ^37^, ^38^, ^39^, ^40^, ^41^, ^42^, ^43^, ^44^, ^45^, ^46^, ^47^, ^48^, ^49^, ^50^, ^51^, ^52^, ^53^, ^54^, ^55^, ^56^, ^57^, ^58^, ^59^, ^60^, ^61^, ^62^, ^63^, ^64^, ^65^, ^66^, ^67^, ^68^ (5644 participants) were included in the review (*Fig*. 1). Characteristics of included studies are summarized in *Table* 1, with additional detail in *Tables S1–S4* (supporting information).

**Table 1:** Summary of trial characteristics (number of studies)

**No. of arms**	Two arms (36), three arms (5), four arms (7)
**Countries**	USA (13), Korea (6), Iran (4), China (3), Denmark (3), Israel (3), UK (2), Poland (2), Turkey (2), Austria (1), Egypt (1), Finland (1), France (1), Germany (1), Pakistan (1), Sweden (1), Taiwan (1), Spain (1), n.r. (1)
**Type of surgery**	Haemorrhoidectomy (7), anorectal (6), hip or knee arthroplasty (5), inguinal hernia (5), mixed surgical populations (5), orthopaedic (5), urogynaecological (5), knee arthroscopy (2), caesarean (1), cataract (1), spinal surgery (2), gastrointestinal (2), lower limb surgery (1), n.r. (1)
**Prevention of PO‐UR** *	
Pharmacological (46)	Anaesthesia (7), morphine avoidance (4), morphine replacement (2), morphine administration (1), NSAID suppository (2), alpha‐blocker (15), μ‐opioid antagonist (2), antispasmodic (1), benzodiazepine (1), cholinergic (4)
Non‐pharmacological (11)	Fluids (3), mobilization (2), acupuncture (1), external bladder stimulator (1), preoperative ultrasound monitoring (1), straight catheterization in recovery room (1), postoperative anal packing (1)
**Treatment of PO‐UR** *	
Pharmacological (12)	Alpha‐blockers (4), cholinergic ± benzodiazepines (4)
Non‐pharmacological (5)	Gauze soaked in warm water/hot pack (3), caffeine (1), posterior tibial nerve stimulation (1), moxibustion (2), infrared radiation (1)
**Secondary outcomes**	UTI (5), length of hospital stay (6), pain (7)

* Some studies are included twice or more as they evaluated multiple interventions. n.r., Not reported; PO‐UR, postoperative urinary retention; NSAID, non‐steroidal anti‐inflammatory drug; UTI, urinary tract infection.

### Description of included studies

The majority of included studies were conducted in surgical populations with a higher risk of developing PO‐UR. Ten studies included only men and seven included only women. Of the 24 studies that included both men and women, 56·6 per cent were men overall. Seven studies did not report on the sex of included participants. Sample size ranged from 30 to 496 (median 99). More studies targeted prevention than considered treatment of PO‐UR (41 *versus* 7), and pharmacological interventions were evaluated more commonly than non‐pharmacological measures. All studies used a definition of PO‐UR that involved some form of active treatment, most commonly catheterization. In addition, some included a measure of bladder volume or a palpable bladder.

Of the 48 included trials, ten^17^ ^23^, ^28^ ^32^, ^38^ ^39^, ^45^ ^55^, ^56^, ^57^ ^67^, ^68^ were considered to be at low risk of bias; eight of these evaluated pharmacological interventions and two evaluated non‐pharmacological interventions. Eleven^18^ ^22^, ^25^ ^29^, ^30^ ^35^, ^40^, ^41^, ^42^, ^43^ ^54^, ^60^ ^66^ were judged at high risk of bias; these studies evaluated pharmacological (7 studies) and non‐pharmacological (3) interventions for the prevention of PO‐UR, and a pharmacological intervention (1 study) for the treatment of PO‐UR. There were 27 studies^15^ ^16^, ^19^, ^20^, ^21^ ^24^, ^26^ ^27^, ^31^ ^33^, ^34^ ^36^, ^37^ ^44^, ^46^, ^47^, ^48^, ^49^, ^50^, ^51^, ^52^, ^53^ ^58^, ^59^ ^61^, ^62^, ^63^, ^64^, ^65^ rated as having some concerns; these studies evaluated pharmacological (16 studies) and non‐pharmacological (5) interventions for the prevention of PO‐UR, and pharmacological (2) and non‐pharmacological (4) interventions for treating PO‐UR.

The main potential limitations of the included studies were methods of randomization/concealment (35 trials) or blinding of participants/carers (23). Fewer studies were found to have concerns regarding missing data (6), failure to blind outcome assessor (8) and selective reporting (8). Overall and domain‐level assessments of risk of bias for each trial are provided in *Appendix S2* (supporting information).

### Pharmacological interventions for prevention of postoperative urinary retention (*Tables S5* and *S6*)

#### *Morphine use in a regional anaesthetic regimen (9 trials, 530 participants) (*Fig. 2 *)*

Reducing exposure to morphine was associated with a reduced risk of PO‐UR. There was moderate‐certainty evidence that avoiding morphine as part of a spinal or epidural anaesthetic regimen (summary OR 0·14, 95 per cent c.i. 0·06 to 0·34; *I* ^2^ = 0 per cent; 3 trials) or adding μ‐opioid antagonists to the anaesthetic (summary OR 0·49, 0·24 to 1·01; *I* ^2^ = 0 per cent; 2 trials) reduced the number of patients with PO‐UR.

**Figure 2:**
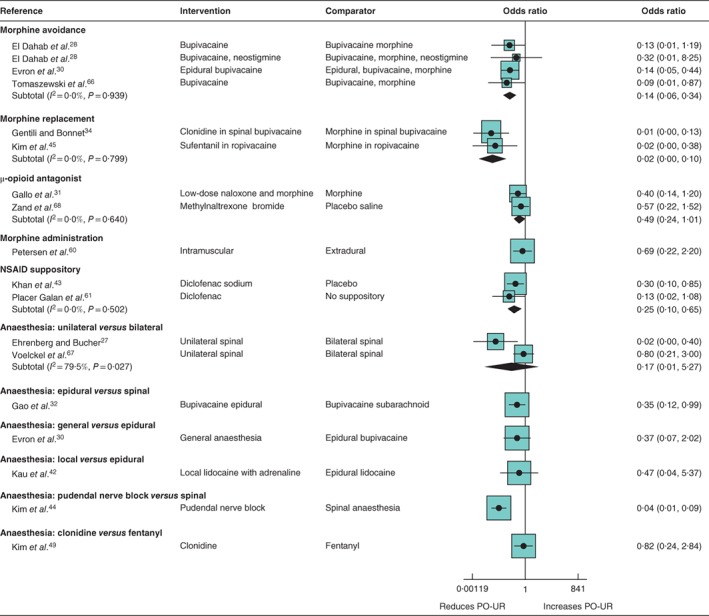
Forest plot comparing anaesthetic modifications for the prevention of postoperative urinary retention. A random‐effects model was used for meta‐analysis where appropriate. Odds ratios are shown with 95 per cent confidence intervals. NSAID, non‐steroidal anti‐inflammatory drug; PO‐UR, postoperative urinary retention.

There was high‐certainty evidence that the incidence of PO‐UR was lower when the anaesthetic regimen included an alternative to morphine (clonidine or sufentanil) for postoperative analgesia (summary OR 0·02, 95 per cent c.i. 0·00 to 0·10; *I* ^2^ = 0 per cent; 2 trials).

There was very low certainty about the effect of intramuscularly administered morphine compared with extradural morphine (OR 0·69, 95 per cent c.i. 0·22 to 2·20; 1 trial)^60^.

Very few studies reported data on secondary outcomes. Single studies each assessed UTI^30^, pain^45^ and duration of hospital stay^31^, and found no evidence for a difference between intervention and control groups.

#### *Non‐steroidal anti‐inflammatory drugs (2 RCTs, 343 participants) (*Fig. 2 *)*

There was moderate‐certainty evidence that diclofenac suppositories reduced PO‐UR in patients undergoing haemorrhoidectomy compared with placebo or no suppository (OR 0·25, 95 per cent c.i. 0·10 to 0·65; 2 trials)^43^ ^61^. One trial^61^ assessed pain and found no effect for diclofenac suppositories on the number of patients requiring postoperative analgesia (OR 0·45, 0·19 to 1·06).

#### *Type of regional anaesthesia (7 RCTs, 795 participants) (*Fig. 2 *)*

There was high‐certainty evidence that the risk of PO‐UR was lower with epidural compared with spinal anaesthesia (0·35, 95 per cent c.i. 0·12 to 0·99; 1 trial)^32^, and that pudendal nerve block was associated with a lower incidence of PO‐UR than spinal anaesthesia (OR 0·04, 0·01 to 0·09; 1 trial)^44^. Moderate‐certainty evidence suggested no difference in the effect of clonidine compared with fentanyl (OR 0·82, 0·24 to 2·84; 1 trial)^49^.

There was very low certainty of no difference in the effect of unilateral compared with bilateral spinal anaesthesia (summary OR 0·17, 95 per cent c.i. 0·01 to 5·27; *I* ^2^ = 79·5 per cent; 2 trials)^27^ ^67^, of general compared with epidural anaesthesia (OR 0·37, 0·07 to 2·02; 1 trial)^30^ and of local compared with epidural anaesthesia (OR 0·47, 0·04 to 5·37; 1 trial)^42^.

Some evidence for increased duration of hospital stay was found when patients received bupivacaine epidurally *versus* subarachnoid bupivacaine (MD 16·08 (95 per cent c.i. 1·40 to 30·76) hours)^32^ and when patients received epidural lidocaine alone *versus* local lidocaine plus adrenaline (epinephrine) (MD 2·28 (1·85 to 2·71) hours)^42^. No evidence for an effect on UTI was found when patients received general anaesthesia compared with epidural anaesthesia. Patients who had a pudendal nerve block reported lower pain ratings than those who received spinal anaesthesia (WMD −2·50, −3·16 to −1·84), and fewer patients required postoperative analgesia (OR 0·20, 0·10 to 0·41)^44^.

#### *Alpha‐blockers (12 RCTs, 1283 participants) (*Fig. 3 *)*

There was high‐certainty evidence that alpha‐blockers were associated with a lower incidence of PO‐UR (summary OR 0·24, 95 per cent c.i. 0·14 to 0·41; *I* ^2^ = 57·6 per cent; 12 trials). Studies compared tamsulosin (5 trials)^16^ ^19^, ^20^ ^57^, ^58^, phenoxybenzamine (3)^30^ ^35^, ^55^, prazosin (3)^26^ ^36^, ^59^, alfuzosin (1 trial)^16^ and dibenzyline (1 trial)^54^ with placebo or no treatment. Two of the phenoxybenzamine trials were restricted to women. All trials of other alpha‐blockers were either restricted to men or did not report on sex. There were no clear differences in effect based on type of alpha‐blocker or sex. There was moderate certainty for no evidence of a difference in effect of tamsulosin compared with alfuzosin (OR 0·74, 0·16 to 3·44; 1 trial)^16^.

**Figure 3:**
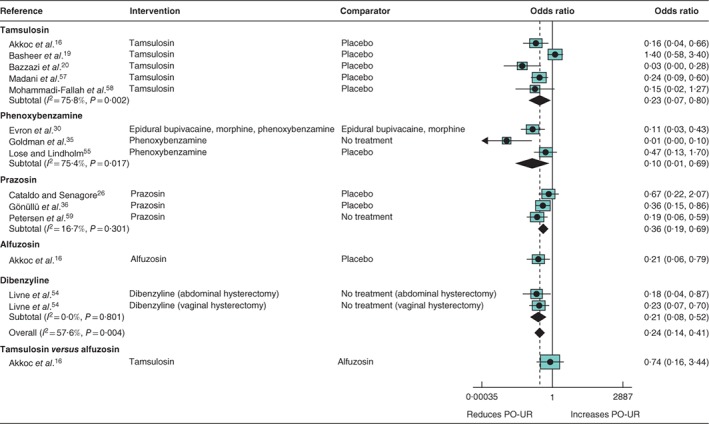
Forest plot comparing alpha‐blocker administration for the prevention of postoperative urinary retention. A random‐effects model was used for meta‐analysis where appropriate. Odds ratios are shown with 95 per cent confidence intervals. PO‐UR, postoperative urinary retention.

No evidence for an effect of alpha‐blockers was found on secondary outcomes of UTI (3 studies), pain (2) or duration of hospital stay (1).

#### *Cholinergic drugs (3 RCTs, 262 participants) (*Fig. 4 *)*

There was low certainty about the effect of cholinergic drugs on PO‐UR (summary OR 0·42, 95 per cent c.i. 0·12 to 1·46; *I* ^2^ = 49·0 per cent; 3 trials)^21^ ^24^, ^28^. Studies compared urecholine (administered orally) with standard care, intramuscular distigmine bromide with saline placebo, and the addition of neostigmine to the anaesthetic regimen.

**Figure 4:**
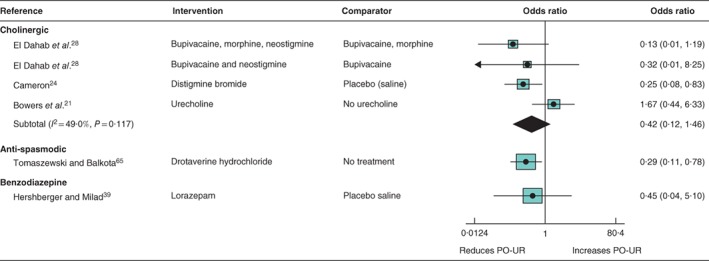
Forest plot comparing pharmacological interventions for prevention of postoperative urinary retention. A random‐effects model was used for meta‐analysis where appropriate. Odds ratios are shown with 95 per cent confidence intervals. PO‐UR, postoperative urinary retention.

#### *Antispasmodic drugs (1 RCT, 201 participants) (*Fig. 4 *)*

There was high‐certainty evidence for a lower incidence of PO‐UR amongst patients given drotaverine hydrochloride intramuscularly after spinal anaesthesia compared with a standard anaesthetic regimen (OR 0·29, 95 per cent c.i. 0·11 to 0·78; 1 trial)^65^.

#### *Benzodiazepines (1 RCT, 90 participants) (*Fig. 4 *)*

There was low certainty regarding the effect of the benzodiazepine lorazepam compared with placebo (OR 0·45, 95 per cent c.i. 0·04 to 5·10; 1 trial)^39^. This trial found no effect for lorazepam on pain scores or hospital stay (h) (MD 1·00, 0·20 to 1·80, and MD 0·80, −0·16 to 1·76, respectively).

### Non‐pharmacological interventions for prevention of postoperative urinary retention (*Tables S5* and *S6*; *Fig*. 5)

**Figure 5:**
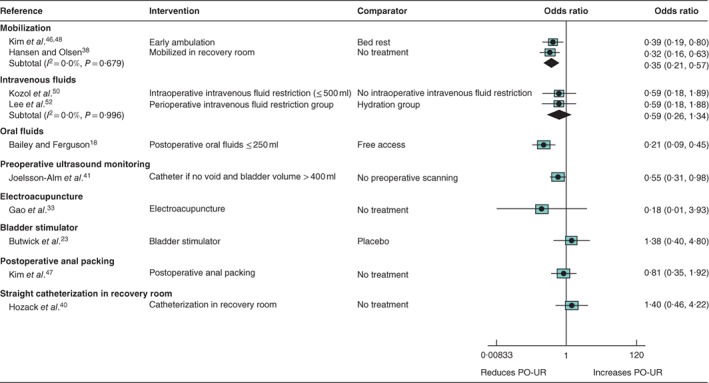
Forest plot comparing non‐pharmacological interventions for prevention of postoperative urinary retention. A random‐effects model was used for meta‐analysis where appropriate. Odds ratios are shown with 95 per cent confidence intervals. PO‐UR, postoperative urinary retention.

#### *Early mobilization (2 RCTs, 305 participants)*

High‐certainty evidence was found for an association between early mobilization and reduced incidence of PO‐UR (summary OR 0·35, 95 per cent c.i. 0·21 to 0·57; *I* ^2^ = 0 per cent; 2 trials)^38^ ^46^, ^48^. There was no evidence for an effect of early mobilization on pain (1 trial)^38^.

#### *Fluid restriction (1 RCT, 496 participants)*

Low‐certainty evidence suggested a lower incidence of PO‐UR amongst patients restricted to 250 ml or less of oral fluids after surgery compared with patients given free access to fluids (OR 0·21, 95 per cent c.i. 0·09 to 0·45; 1 trial)^18^. There was moderate certainty for no evidence of an effect of intravenous fluid restriction (summary OR 0·59, 0·26 to 1·34; *I* ^2^ = 0 per cent; 2 trials)^50^ ^52^.

#### *Other approaches*

There was low‐certainty evidence of reduced PO‐UR in patients assessed with frequent preoperative ultrasound scanning and catheterized when the bladder volume exceeded 400 ml, compared with patients with no preoperative scanning (OR 0·55, 95 per cent c.i. 0·31 to 0·98; 1 trial)^41^. Trials investigating other non‐pharmacological interventions, including electroacupuncture^33^, external bladder stimulator^23^, postoperative anal packing after haemorrhoidectomy^47^ and straight catheterization in the recovery room^40^, found no evidence for a reduction in PO‐UR.

There was no evidence for an effect of bladder stimulator^23^ on pain, for preoperative ultrasound monitoring^41^ or straight catheterization in recovery room^40^ on UTI, or for preoperative ultrasound monitoring^41^ on duration of hospital stay.

### Pharmacological treatment of postoperative urinary retention (*Tables S5* and *S6*; *Fig*. 6)

**Figure 6:**
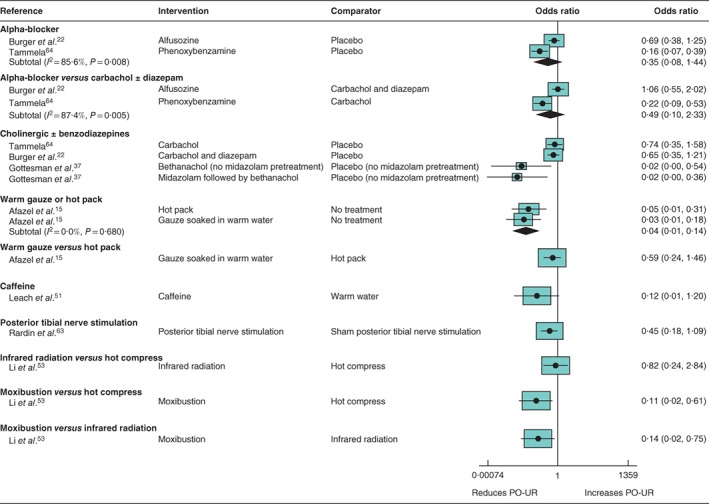
Forest plot comparing pharmacological and non‐pharmacological interventions for treatment of postoperative urinary retention A random‐effects model was used for meta‐analysis where appropriate. Odds ratios are shown with 95 per cent confidence intervals. PO‐UR, postoperative urinary retention.

#### *Alpha‐blockers (2 RCTs, 543 participants)*

There was very low certainty regarding the effect of phenoxybenzamine or alfuzosin compared with placebo (summary OR 0·35, 95% CI 0·08 to 1·44; 2 trials)^22^ ^64^. The same trials provided evidence of very low certainty about the effect of phenoxybenzamine *versus* carbachol or alfuzosin *versus* carbachol and diazepam (summary OR 0·49, 0·10 to 2·33).

#### *Benzodiazepines and cholinergic drugs (4 RCTs, 325 participants)*

There was no evidence of a reduction in PO‐UR among patients given carbachol, with or without diazepam, compared with placebo (OR 0·74, 95 per cent c.i. 0·35 to 1·58, and OR 0·65, 0·35 to 1·21)^22^ ^64^. There was no evidence for an effect of midazolam, although fewer patients developed PO‐UR when they were subsequently rerandomized to receive bethanechol (OR 0·02, 0·00 to 0·54, and OR 0·02, 0·00 to 0·36, respectively)^37^.

### Non‐pharmacological treatment of postoperative urinary retention (4 trials) (*Tables S5* and *S6*; *Fig*. 6)

Interventions for the treatment of urinary retention were evaluated only in single trials. High‐certainty evidence was found for a beneficial effect of applying a hot pack or gauze soaked in warm water to the suprapubic region (OR 0·04, 95 per cent c.i. 0·01 to 0·14; 1 trial)^15^. The same trial provided moderate certainty that there was no difference of effect between the two interventions. There was also moderate‐certainty evidence for reduced PO‐UR amongst patients given posterior tibial nerve stimulation (PTNS) compared with sham PTNS (OR 0·45, 0·18 to 1·09; 1 trial)^63^. There was low‐certainty evidence that a cup of warm coffee reduced PO‐UR compared with warm water (OR 0·12, 0·01 to 1·20; 1 trial)^51^, and for no effect of infrared radiation compared with a hot compress (OR 0·82, 0·24 to 2·84; 1 trial)^53^. This trial also provided moderate‐certainty evidence that moxibustion was associated with a lower incidence of PO‐UR compared with either a hot compress or infrared radiation (OR 0·11, 0·02 to 0·61, and OR 0·14, 0·02 to 0·75, respectively).

## Discussion

This systematic review summarizes the effectiveness of interventions evaluated for the prevention or treatment of PO‐UR. The majority of the 48 RCTs evaluated preventive interventions. Based on GRADE assessments, there was high‐certainty evidence to suggest that the following may help reduce the risk of patients developing PO‐UR after surgery: replacing morphine in the anaesthetic regimen; using alpha‐blockers or the antispasmodic drotaverine; early postoperative mobilization; and epidural anaesthesia or pudendal nerve blocks rather than spinal anaesthesia. The number of patients who would need to receive the intervention for one case of urinary retention to be prevented ranged from 2 (95 per cent c.i. 2 to 3) for morphine replacement to 9 (7 to 30) for drotaverine. There was some evidence that avoiding morphine in the anaesthetic regimen, diclofenac suppositories (NSAID), μ‐opioid antagonists, oral fluid restriction and preoperative ultrasound monitoring were also associated with a lower risk of PO‐UR, but the evidence here was less certain. The number of patients that would need to receive these intervention**s** for one case of PO‐UR to be prevented ranged from 4 (4 to 6) for morphine avoidance to 11 (9 to 25) for NSAIDs. For patients who developed PO‐UR after surgery, there was high‐certainty evidence that a hot pack or gauze soaked in warm water reduced the need for catheterization, with two patients needing this treatment to resolve one case of urinary retention. There was also evidence that moxibustion, posterior tibial nerve stimulation and caffeinated drinks could help to treat PO‐UR, although the evidence was less certain.

There was no evidence that the following could help to reduce the risk of PO‐UR: use of an external bladder stimulator, postoperative anal packing (after haemorrhoidectomy), cholinergic drugs, lorazepam (benzodiazepine) and electroacupuncture, intramuscularly administered morphine (*versus* extradural administration), unilateral anaesthesia (*versus* bilateral anaesthesia), general or local anaesthesia (*versus* epidural anaesthesia) and straight catheterization in the recovery room. For treatment of PO‐UR, there was no evidence to support the use of alpha‐blockers or cholinergic drugs with or without benzodiazepines.

This review had a number of strengths and limitations. Cochrane recommendations for conducting systematic reviews^8^ were followed. A highly sensitive search strategy was developed and performed by an independent information specialist, to identify as many relevant studies as possible and reduce the risk of publication bias. Searches were not limited by language or publication status. Supplementary internet searches were undertaken to identify grey literature, including ongoing and completed clinical trials. However, it was not possible to contact study authors to request unavailable data. In addition, owing to resource limitations, it was not possible to translate studies where reviewers were unable to identify whether the study fully met the inclusion criteria. Although one study^53^ evaluating the effects of moxibustion and infrared radiation was identified, the search did not include Chinese databases in which studies of similar interventions are more likely to be reported. A review of studies evaluating moxibustion as an intervention for treating PO‐UR^69^ included 19 studies identified from Chinese sources; these were not targeted in the search strategy due to difficulties in accessing and interpreting this evidence.

To minimize bias and errors, all stages of the review were performed independently by two reviewers, including a public contributor who had experienced PO‐UR. All included RCTs were assessed using the new Cochrane risk‐of‐bias 2.0 tool^10^ ^11^. This identified a number of methodological weaknesses in the included trials, particularly in relation to methods of treatment allocation and blinding of participants and study personnel. GRADE^14^ was used to assess the quality of the overall body of evidence. This allowed for systematic and transparent ratings summarizing the quality of evidence for each intervention evaluated, taking into account both the strength of the association (magnitude of effect and precision) and the risk of bias in the included studies, inconsistency (heterogeneity), publication bias and applicability to the research question. Reasons for downgrading evidence, where appropriate, are shown in *Table S5* (supporting information).

There were a number of limitations in the reviewed studies. A wide variety of definitions for urinary retention were used. Although the majority of trials defined PO‐UR by the need for a urinary catheter, catheterization protocols varied between studies. Catheter use can be considered a strong proxy measure of PO‐UR, but for studies where it was not stated explicitly that catheterization was performed as treatment for urinary retention, it must be noted that there could be other reasons for catheterization, including postoperative fluid balance monitoring or poor mobility^70^. The surgical population varied across studies, as did surgical and anaesthetic techniques. Included studies assessed a wide range of interventions, and analyses were stratified based on the aim (prevention or treatment), type (pharmacological or non‐pharmacological) and category of intervention. Very few studies evaluated the same interventions: usually a maximum of three per comparison. The only comparisons involving a large number of studies were related to alpha‐blockers, which were evaluated in 12 trials. Within this category, several different types of alpha‐blocker were evaluated, with a maximum of five studies evaluating the same intervention. Thus there were insufficient data to conduct a sensitivity analysis, which would have provided information on whether the effectiveness of interventions varied based on factors such as sex, type of surgery, intervention dose, ethnicity or risk of bias. Most were two‐arm trials with a placebo or control group (no treatment), but some studies included active comparisons and multiple groups comparing more than one category of intervention. This resulted in a wide variety of comparisons, some consisting of single studies. Studies assessing interventions of the same category often differed in the method or timing of administration. This resulted in considerable heterogeneity amongst the included studies and meta‐analysis was inappropriate. Where heterogeneity was present in a meta‐analysis, GRADE assessments were downgraded to reflect reduced certainty in the evidence. The most common reason for downgrading was confidence intervals that included the null, followed by risk of bias.

There are two existing reviews related to PO‐UR. A Cochrane systematic review^71^ evaluated drugs for the treatment of urinary retention after surgery. This review^71^ identified seven studies evaluating the effect of cholinergic agents, alpha‐blockers, sedatives and prostaglandin (alone or in combinations) on PO‐UR. Three of the studies did not meet the inclusion criteria for the present review: one measured PO‐UR on postoperative day nine, 72 h after catheter removal, and two were conducted in populations of women with stress urinary incontinence. The review concluded that there was ‘little trial‐based evidence that could shed light on the effectiveness or otherwise of alpha‐blockers or sedatives in the treatment of post‐operative urinary retention’. This is consistent with the findings of the present review with regard to treatment of PO‐UR. The other review article^72^ was restricted to trials of alpha‐blockers for prevention of PO‐UR. It included 15 RCTs and reported a significantly reduced risk of PO‐UR, consistent with the findings of the present review. There are also two published Cochrane protocols which are yet to be completed, one aimed at reviewing the evidence on drugs for the prevention of PO‐UR (2013)^73^ and the other on non‐drug treatments forPO‐UR (2015)^6^.

Large, robust and well designed RCTs are needed to confirm the effects of the interventions studied in this review, and to inform the design of an effective prevention and treatment protocol for surgical patients at risk of developing PO‐UR. Trials should follow CONSORT reporting standards^74^ to ensure appropriate methods for minimizing risk of bias, along with the perspectives of patients and economic evaluation.

## Supporting information

Appendix S1 Search strategy
Table S1 Characteristics of included studies: pharmacological prevention
Table S2 Characteristics of included studies: non‐pharmacological prevention
Table S3 Characteristics of included studies: pharmacological treatment
Table S4 Characteristics of included studies: non‐pharmacological treatment
Table S5 Summary estimates for the effects of interventions on the incidence of postoperative urinary retention together with GRADE ratings for certainty of the evidence
Table S6 Secondary outcomes
Appendix S2 Risk of bias by type of intervention
