# Supplementary material for: Systematic review of interventions for the prevention and treatment of postoperative urinary retention
Source: BJS Open. 2018 Nov 19;3(1):11–23. doi: 10.1002/bjs5.50114 (PMC6354194; doi:10.1002/bjs5.50114)
Supplement: Supplementary file 1 — Appendix S1 Search strategy Table S1 Characteristics of included studies: pharmacological prevention Table S2 Characteristics of included studies: non‐pharmacological prevention Table S3 Characteristics of included studies: pharmacological treatment Table S4 Characteristics of included studies: non‐pharmacological treatment Table S5 Summary estimates for the effects of interventions on the incidence of postoperative urinary retention together with GRADE ratings for certainty of the evidence Table S6 Secondary outcomes Appendix S2 Risk of bias by type of intervention [file BJS5-3-11-s001.docx]

**BJS5_50114**

# **Systematic review of interventions for the prevention and treatment of postoperative urinary retention**

**J. Jackson, P. Davies, N. Leggett, M. Nugawela, L. J. Scott, V. Leach, A. Richards, A. Blacker, P. Abrams, J. Sharma, J. Donovan and P. Whiting**

# **Appendix S1 Search strategy**

Database: Ovid MEDLINE(R) <1946 to present>

Search Strategy:

--------------------------------------------------------------------------------

1 Postoperative Complications/ur [Urine] (235)

2 ((void or voiding or postvoid or bladder emptying or micturation) adj3 (postsurg$ or postoperativ$ or post-surg$ or post-operativ$ or intraoperativ$ or intra-operativ$ or perioperativ$ or peri-operativ$ or peroperativ$ or per-operativ$)).ti,ab. (520)

3 (urination disorders/ or urinary bladder/ or urination/) and (perioperative care/ or postoperative care/ or intraoperative care/ or exp intraoperative period/ or exp postoperative period/ or perioperative period/ or postoperative complications/) (4423)

4 or/1-3 (5070)

5 Urinary retention/ (3793)

6 urinary bladder/de or urinary tract/de (5830)

7 ((urin$ or bladder$ or void or voiding) adj6 (retent$ or retain$)).ti,ab. (9557)

8 ((void or voiding or micturation) adj3 (problem$ or dysfunction$ or difficult$ or efficiency)).ti,ab. (3231)

9 (bladder adj3 empty$ adj3 (incomplet$ or problem$ or difficult$ or dysfunction$)).ti,ab. (299)

10 (bladder adj2 (distention or overdistention)).ti,ab. (309)

11 (bladder function adj3 (impair$ or disturb$ or disrupt$)).ti,ab. (122)

12 or/5-11 (19909)

13 exp postoperative period/ (42872)

14 postoperative complications/ (303188)

15 postoperative care/ (53771)

16 perioperative care/ (9762)

17 perioperative period/ or intraoperative care/ or exp intraoperative period/ (32800)

18 (surgery patient$ or postsurg$ or postoperativ$ or peroperativ$ or per-operativ$ or perioperativ$ or peri-operativ$ or post-surg$ or post-operativ$ or intraoperativ$ or intra-operativ$).ti,ab. (559963)

19 ((after or scheduled for or following or during or undergoing or undergone) adj6 (surg$ or operation$ or operativ$ or arthroplast$ or hip replacement$ or knee replacement$ or herniorraph$ or hernia repair$ or hysterectom$)).ti,ab. (523098)

20 or/13-19 (1088586)

21 *analgesia, epidural/ or *anesthesia/ or exp *anesthesia, conduction/ or exp *anesthesia, general/ (114262)

22 analgesia, epidural/ae or anesthesia/ae or exp anesthesia, conduction/ae or exp anesthesia, general/ae (21741)

23 ((local or general or regional or epidural or spinal or systemic) adj2 (an?esthetic$ or an?esthesia or analgesia)).ti,ab. (98148)

24 ((epidural or intrathecal or spinal) adj2 (opioid$ or diamorphine or morphine or sufentanil)).ti,ab. (3856)

25 ((epidural or spinal or peripheral nerve or conduction) adj2 block$).ti,ab. (9007)

26 or/21-25 (183596)

27 12 and (20 or 26) (4994)

28 4 or 27 (9308)

29 letter/ (903410)

30 editorial/ (394702)

31 news/ (174532)

32 exp historical article/ (364962)

33 Anecdotes as topic/ (4684)

34 comment/ (652445)

35 case report/ (1765183)

36 (letter or comment$).ti. (108331)

37 animals/ not humans/ (4157323)

38 exp Animals, Laboratory/ (756753)

39 exp Animal Experimentation/ (7775)

40 exp Models, Animal/ (452337)

41 exp rodentia/ (2825498)

42 (rat or rats or mouse or mice or cat or cats or dog or dogs).ti. (1334758)

43 or/29-42 (8383001)

44 28 not 43 (7734)

45 meta-analysis/ (61714)

46 meta-analysis as topic/ (14493)

47 (meta analy$ or metaanaly$ or metanaly$ or meta regression).ti,ab. (87248)

48 ((systematic$ or evidence$) adj2 (review$ or overview$)).ti,ab. (100533)

49 (reference list$ or bibliograph$ or hand search$ or manual search$ or relevant journals).ab. (29815)

50 (search strategy or search criteria or systematic search or study selection or data extraction).ab. (32283)

51 (search$ adj4 literature).ab. (35846)

52 (medline or pubmed or cochrane or embase or psychlit or psyclit or psychinfo or cinahl or science citation index or bids or cancerlit).ab. (112596)

53 cochrane.jw. (11820)

54 ((multiple treatment$ or indirect or mixed) adj2 comparison).ti,ab. (1127)

55 or/45-54 (249456)

56 randomized controlled trial.pt. or randomized controlled trial/ or Randomized Controlled Trials as Topic/ (502869)

57 controlled clinical trial.pt. (90121)

58 randomi#ed.ab. (402420)

59 placebo.ab. (166590)

60 randomly.ab. (242940)

61 trial.ti,ab. (411331)

62 groups.ab. (1519254)

63 clinical trials as topic.sh. (174976)

64 or/56-63 (2390931)

65 44 and (55 or 64) (1913)

## Table S1 Characteristics of included studies: pharmacological prevention

| **Study**  **Country** | **Surgical population** | **Intervention arms** | **Patients**  **(number randomised, mean age (years), %male)** | **PO-UR definition** | **Secondary outcomes** |
| --- | --- | --- | --- | --- | --- |
| Akkoc 2016^16^  Turkey | Inguinal; penile; scrotal; perineal  (n=180) | 1. Tamsulosin | N=60, 36.3, 100% | Painful, palpable mass in suprapubic area; inability to void within 12 hours post-op, confirmed by emptying more than 500mL of urine by catheterization. |  |
|  |  | 2. Alfuzosin ER (extended release) | N=60, 36.6, 100% |  |  |
|  |  | 3. Placebo | N=60, 34.9, 100% |  |  |
| Basheer 2017^19^  USA | Spinal surgery  (n=98) | 1. Tamsulosin | N=49, 57.7, 100% | Straight catheterization performed for patients with PVR greater than 250 ml every 6 h. | Pain |
|  |  | 2. Placebo | N=49, 57.0, 100% |  |  |
| Bazzazi 2014^20^  NR | Cataract  (n=67) | 1. Tamsulosin | N=32, 68.2, 100% | Patients unable to voluntarily void urine or asking for help with urination underwent urinary catheterization. |  |
|  |  | 2. Placebo | N=35, 71.4, 100% |  |  |
| Bowers 1987^21^  USA | Anorectal  (n=108) | 1. Urecholine, 50 mg | N=26^**^, NR, NR% | Patients catheterized only when they had symptomatic, distended bladders. |  |
|  |  | 2. Urecholine, 25 mg | N=26^**^, NR, NR% |  |  |
|  |  | 3. Urecholine, 5 mg | N=29^**^, NR, NR% |  |  |
|  |  | 4. No Urecholine | N=27^**^, NR, NR% |  |  |
| Cameron 1966^24^  UK | Vaginal/hysterectomy  (n=54) | 1. Distigimine bromide (Ubretid) | N=24^**^, NR, 0% | Required catheterization |  |
|  |  | 2. Placebo (saline) | N=30^**^, NR, 0% |  |  |
| Cataldo 1991^26^  USA | Anorectal  (n=51) | 1. Prazosin | N=26, NR, NR% | Patients catheterised only if failed to void eight hours after surgery and were distended or uncomfortable. | Pain rated on a scale from 0 to 3 (0 = no pain, 1 = mild pain, 2 = moderate pain, 3 = worst pain ever |
|  |  | 2. Placebo | N=25, NR, NR% |  |  |
| Ehrenberg 2010^27^  Germany | Not reported  (n=240) | 1. Isobaric 0.5% bupivacaine for bilateral spinal | N=114^**^, NR, NR% | Patients requiring catheterization |  |
|  |  | 2. Hyperbaric 0.5% bupivacaine for unilateral spinal | N=126^**^, NR, NR% |  |  |
| El Dahab 2011^28^  Egypt | Inguinal Hernia  (n=100) | 1. Bupivacaine plus morphine and neostigmine | N=25, 37, 100% | Patients needing urinary catheterization. Patients instructed to void when feel desires or 4 h post operatively when no desire felt, if patient failed to void we waited one more hour. Urinary catheter inserted if no volitional voiding and U/S bladder volume ≥400 ml. |  |
|  |  | 3. Bupivacaine plus neostigmine | N=25, 35, 100% |  |  |
|  |  | 4. Bupivacaine plus morphine | N=25, 35, 100% |  |  |
|  |  | 2. Bupivacaine only | N=25, 36, 100% |  |  |
| Evron 1984^30^  Evron 1984^29^*  Israel | Caesarean  (n=150) | 1. Epidural anaesthesia (Bupivacaine 0.5%) plus morphine | N=40^**^, NR, 0% | Sensation of a full bladder or palpable distended bladder, if no spotaneous voiding after this then the bladder was emptied by catheterisation | UTI |
|  |  | 2. Epidural anaesthesia (Bupivacaine 0.5%) plus morphine plus phenoxybenzamine | N=30^**^, NR, 0% |  |  |
|  |  | 3. Epidural anaesthesia (Bupivacaine 0.5%) | N=40^**^, NR, 0% |  |  |
|  |  | 4. General anaesthesia | N=40^**^, NR, 0% |  |  |
| Gallo 2008^31^  USA | Orthopaedic  (n=97) | 1. Morphine with Naloxone | N=52, 55, 61.5% | Catheterisation. | Length of hospital stay |
|  |  | 2. Morphine | N=45, 55.1, 62.2% |  |  |
| Gao 2017^32^  China | Lower limb surgery  (n=120) | 1. 3ml 0.5% Bupivacaine Subarachnoid | N=30, 39.4, 43.3% | Indwelling catheter when patients with postoperative bladder filled with urine, diagnosed by ultrasound and cannot be properly discharged. | Length of hospital stay |
|  |  | 2. 3ml 0.25% Bupivacaine Subarachnoid | N=30, 41.1, 50% |  |  |
|  |  | 3. 3ml 0.375% Bupivacaine Subarachnoid | N=30, 43.5, 46.7% |  |  |
|  |  | 4. 6-7ml 0.5% bupivacaine epidural | N=30, 38.1, 56.7% |  |  |
| Gentili 1996^34^  France | Hip surgery  (n=40) | 1. Clonidine | N=20, 64.80, 80% | Bladder distension, inability to void/persistent distention after micturition, treated with naloxone. |  |
|  |  | 2. Morphine | N=20, 61.20, 65% |  |  |
| Goldman 1988^35^  Israel | Inguinal Hernia  (n=102) | 1. Prophylactic Phenoxybenzamine | N=58, NR, NR% | Urinary retention treated with phenoxybenzamine or catheterization |  |
|  |  | 2. No Phenoxybenzamine | N=44, NR, NR% |  |  |
| Gonullu 1999^36^  Turkey | Inguinal Hernia  (n=156) | 1. Prazosin | N=84, 39, 100% | Palpable mass in the suprapubic area, discomfort, and failed to pass urine within 10 hours after operation, despite sufficient fluid intake. A warm bag containing water (40 to 45 degrees C) was applied to all patients with difficulty in passing urine as a first-line therapy. Patients were also encouraged to stand up and walk. Results section implies that some, but not all, patients with urinary retention were catheterised. |  |
|  |  | 2. Placebo | N=72, 36, 100% |  |  |
| Hershberger 2003^39^  USA | Gynaecology  (n=97) | 1. Lorazepam | N=49, 36, 0% | Patients catheterised if unable to void after 6-8 hours after surgery | Length of hospital stay |
|  |  | 2. Placebo saline | N=48, 35, 0% |  | Pain (VAS) |
| Kau 2003^42^  Taiwan | Hemorrhoidectomy  (n=112) | 1. Lidocaine (Epidural) | N=55, 40.8, 60% | Need for catheterisation | Length of hospital stay |
|  |  | 2. Lidocaine with epinephrine (local) | N=57, 42, 59.6% |  |  |
| Khan 2015^43^  Pakistan | Hemorrhoidectomy  (n=186) | 1. Diclofenac sodium suppository | N=93, 35.8, 82.8% | Foley catheter for urinary retention in the first 24 hrs post surgery |  |
|  |  | 2. Placebo suppository | N=93, 35.9, 81.7% |  |  |
| Kim 2006^45^  Korea | Gastrectomy  (n=60) | 1. Sufentanil in ropivacaine | N=30, 63, 50% | Serious or major micturition problems (according to classification defined by Vercauteren et al), requiring indwelling bladder catheterization. | Pain (VAS resting) |
|  |  | 2. Morphine in ropivacaine | N=30, 61, 56.7% |  | Pain (VAS coughing) |
| Kim 2000^44^  Korea | Hemorrhoid surgery  (n=163) | 1. Pudendal nerve block | N=81, NR, 54.9% | Urinary catheterization needed | Pain (# required analgesics) |
|  |  | 2. Spinal anaesthesia | N=82, NR, 50.6% |  | Pain (VAS) |
| Kim 2001^49^  Korea | Hemorrhoidectomy  (n=40) | 1. Clonidine | N=20, 37.5, NR% | Reports "number of patients using a urinary catheter to void" |  |
|  |  | 2. Fentanyl | N=20, 39, NR% |  |  |
| Livne 1983^54^  Israel | Hysterectomy  (n=155) | 1. Abdominal hysterectomy Dibenzyline | N=51^**^, 47.9, 0% | Foley urethral catheter used for drainage and left in place for 24 to 36 hours if bladder overdistension (identified by physical examination) or patient did not pass urine during the first 24 hours post-op |  |
|  |  | 2. Vaginal hysterectomy Dibenzyline | N=30^**^, 57, 0% |  |  |
|  |  | 3. Abdominal hysterectomy control | N=48^**^, 48.7, 0% |  |  |
|  |  | 4. Vaginal hysterectomy control | N=26^**^, 57.3, 0% |  |  |
| Lose 1985^55^  Denmark | Uterovaginal prolapse  (n=41) | 1. Phenoxybenzamine | N=20^**^, 65, 0% | Transrethral catheterisation if voiding inability appeared. | UTI |
|  |  | 2. Placebo | N=21^**^, 66, 0% |  | Length of hospital stay |
| Madani 2014^57^  Madani 2011^56^  Iran | Inguinal herniorrhaphy, varicocelectomy or scrotal surgeries  (n=232) | 1. Tamsulosin | N=118, 27.6, 100% | Painful and palpable mass in suprapubic area and unable to void during first 24 hours after surgery. Diagnosis confirmed by emptying of more than 400ml of urine by catheterization. |  |
|  |  | 2. Placebo | N=114, 27.7, 100% |  |  |
| Mohammadi-Fallah 2012^58^  Iran | Inguinal Hernia  (n=80) | 1. Tamsulosin (0.4mg) | N=40, 66.2, 100% | Bladder catheterization if palpable mass in suprapubic area, discomfort and failed to pass urine within 24 hours after operation despite fluid intake and when conservative efforts such as warming the suprapubic region and encouraging the patient to stand up and walk were unsuccessful |  |
|  |  | 2. Placebo | N=40, 63.3, 100% |  |  |
| Petersen 1982^60^  Denmark | Gastrointestinal  (n=56) | 1. Morphine (extradural) | N=36, 42.6, 43.7% | Patients with sensation and discomfort of a full bladder or palpable distended bladder encouraged to void during the following hour (all required treatment) |  |
|  |  | 2. Morphine (intramuscular) | N=20, 48.1, 60% |  |  |
| Petersen 1991^59^  USA | Hip or Knee arthroplasty  (n=60) | 1. Prazosin hydrochloride | N=28, 63.8, 100% | Inability to spontaneously void when the bladder became distended. Treated with intermittent catheterization every six hours as necessary. | UTI |
|  |  | 2. No prazosin hydrochloride | N=32, 66.7, 100% |  |  |
| Placer Galan 2008^61^  Spain | Haemorrhoidectomy  (n=157) | 1. Diclofenac anal suppository | N=73, 55, 32.9% | Requiring placement of a urinary catheter. | Pain (# required analgesics) |
|  |  | 2. No suppository | N=84, 52, 34.5% |  |  |
| Tomaszewski 2014^66^  Poland | Orthopaedic  (n=30) | 1. Bupivacaine 0.5% | N=16^**^, 26.9, 50% | Need for urinary catheterisation |  |
|  |  | 2. Bupivacaine 0.5% plus morphine | N=14^**^, 26.9, 92.9% |  |  |
| Tomaszewski 2015^65^  Poland | Orthopaedic  (n=228) | 1. Drotaverine hydrochloride | N=114, Median 29, 75% | Discomfort or pain in the lower abdomen treated by urinary catheterisation. |  |
|  |  | 2. Control | N=114, Median 28, 53.5% |  |  |
| Voelckel 2009^67^  Austria | Knee arthroscopy  (n=40) | 1. Bi-lateral spinal anaesthesia | N=20, 56.5, 65% | Inability to void spontaneously and urinary bladder volume >500ml (bladder scan) requiring subsequent catheterization. |  |
|  |  | 2. Uni-lateral spinal anaesthesia | N=20, 54.5, 55% |  |  |
| Zand 2015^68^  Asadi 2012^17^  Iran | Orthopaedic  (n=67) | 1. Methylnaltrexone Bromide | N=33^**^, 33, 87.9% | Inability to urinate for 0.5 hour despite the urge for urination and drainage of urine after bladder catheterization, or inability to urinate for 12 hours after surgery, without feeling the need to urinate, and drainage of at least 600 mL of urine after bladder catheterization |  |
|  |  | 2. Placebo saline | N=34^**^, 38, 76.5% |  |  |
|  |  |  |  |  |  |

*Abstract only; includes subset of patients from Evron^32^. **Number treated.

**Table S2** **Characteristics of included studies: non-pharmacological prevention**

| **Study**  **Country** | **Surgical population** | **Intervention arms** | **Patients**  **(number randomised, mean age (years), %male)** | **PO-UR definition** | **Secondary outcomes** |
| --- | --- | --- | --- | --- | --- |
| Bailey 1976^18^  USA | Anorectal  (n=496) | 1. Oral fluids limited to 250ml postoperatively | N=228**, 47, 52% | Patients catheterised only for bladder distension |  |
|  |  | 2. Free access to oral fluids | N=268**, 47, 63% |  |  |
| Butwick 2003^23^  UK | Knee Replacement  (n=43) | 1. External bladder stimulator | N=22, 69, 72.7% | Need for catheterisation | Pain |
|  |  | 2. Placebo | N=21, 72, 76.2% |  |  |
| Gao 2014^33^  China | Knee arthroscopy  (n=61) | 1. Electroacupuncture | N=31, 55, 32.3% | Once the bladder volume greater than 600ml indwelling urethral catheterization was perfomed |  |
|  |  | 2. No intervention | N=30, 53, 30% |  |  |
| Hansen 2015^38^  Denmark | Disc herniation  (n=152) | 1. Mobilised in recovery room | N=76, Median 49, 55.20% | In-out-catheterisation if bladder volume > 400 ml, after an attempt of voiding (regardless of wheter they had passed urine) | Pain (VAS) |
|  |  | 2. Not mobilised in recovery room | N=76, Median 49, 47.8% |  |  |
| Hozack 1988^40^  Carpiniello 1988^25^  USA | Hip or Knee arthroplasty  (n=54) | 1. Straight catheterization in recovery room | N=31**, 73, 0% | Need for catheterisation after recovery room | UTI |
|  |  | 2. No catheterization in recovery room | N=23**, 70, 0% |  |  |
| Joelsson-Alm 2012^41^  Sweden | Orthopaedic  (n=300) | 1. Pre-operative ultrasound monitoring | N=150, Median 72, 48% | Bladder distension (ultrasound bladder volume >500 ml), or subsequent voided/ catheterized urine volumes. Patients with indwelling Foley catheters measured after withdrawal of the catheter. | UTI |
|  |  | 2. No pre-op scanning | N=150, Median 75, 30% |  | Length of hospital stay |
| Kim 1999^46^  Kim 1999^48^  Korea | Anorectal  (n=153) | 1. Early ambulation | N=75, 35.3, 62.7% | Catheterisation |  |
|  |  | 2. Bed rest | N=78, 35.6, 60% |  |  |
| Kim 2000^47^  Korea | Hemorrhoidectomy  (n=119) | 1. Post-op anal packing | N=62, 37.7, 59.7% | If urinary retention occurred, urinary catheterisation was done temporarily |  |
|  |  | 2. No post-op anal packing | N=57, 35.1, 63.2% |  |  |
| Kozol 1992^50^  USA | Inguinal Hernia  (n=113) | 1. Intraoperative IV fluid restriction (<=500ml) | N=53, 54, 100% | Urinary retention was defined as the requirement of bladder catheterization. Patients having a spontaneous urine output of less than 100 ml within the first 8 hr were catheterized. In addition, any patients with lower abdominal discomfort and a palpable bladder were catheterized. |  |
|  |  | 2. No intraoperative IV fluid restriction | N=60, 54, 100% |  |  |
| Lee 1999^52^  Korea | Anorectal  (n=80) | 1. Restriction group | N=37, 40.1, 5% | Patients catheterised |  |
|  |  | 2. Hydration group | N=43, 40.9, 44.1% |  |  |

**Table S3** **Characteristics of included studies: pharmacological treatment**

| **Study**  **Country** | **Surgical population** | **Intervention arms** | **Patients**  **(number randomised, mean age (years), %male)** | **PO-UR definition** | **Secondary outcomes** |
| --- | --- | --- | --- | --- | --- |
| Burger 1997^22^  America | Surgical  (n=249) | 1. Alfusozine | N=82, 59.5, 68.2% | If no spontaneous miction occurred within 2 hours after taking the randomly administered medication, patients were catheterized. |  |
|  |  | 2. Carbachol & Diazepam | N=72, 60.1, 62.5% |  |  |
|  |  | 3. Placebo | N=95, 59.4, 57.8% |  |  |
| Gottesman 1989^37^  USA | Hemorrhoidectomy  (n=76) | 1. Midazolam followed by bethanachol | N=9, NR, NR% | Patients who failed to void proceded to second (randomised) treatment intervention, or were catheterised. |  |
|  |  | 2. Bethanachol (no midazolam pre-treatment) | N=9, NR, NR% |  |  |
|  |  | 3. Midazolam followed by placebo | N=4, NR, NR% |  |  |
|  |  | 4. Placebo (no midazolam pre-treatment) | N=16, NR, NR% |  |  |
| Tammela 1986^64^  Finland | Various surgical  (n=165) | 1. Carbachol | N=55, 54.8, 56.4% | Patients were recatheterized if spontaneous voiding still was not possible and the bladder was full. |  |
|  |  | 2. Phenoxybenzamine | N=55, 59.1, 62.5% |  |  |
|  |  | 3. Placebo | N=55, 57.4, 57.7% |  |  |

**Table S4** **Characteristics of included studies: non-pharmacological treatment**

| **Study**  **Country** | **Surgical population** | **Intervention arms** | **Patients**  **(number randomised, mean age (years), %male)** | **PO-UR definition** | **Secondary outcomes** |
| --- | --- | --- | --- | --- | --- |
| Afazel 2014^15^  Iran | Orthopedic/urologic/general  (n=126) | 1. Hot pack | N=42, 41, 100% | If UR was not relieved after 20 minutes of beginning the intervention, urinary catheterization was performed immediately. |  |
|  |  | 2. Gauze soaked in warm water | N=42, 45.3, 100% |  |  |
|  |  | 3. No intervention | N=42, 43.4, 100% |  |  |
| Leach 2013^51^  USA | Hip or Knee arthroplasty  (n=30) | 1. Caffeine | N=16, NR, NR% | Patients requiring catheterization |  |
|  |  | 2. Warm water | N=14, NR, NR% |  |  |
| Rardin 2011^63^  Rardin 2006^62^  USA | Urogynaecologic  (n=84) | 1. Posterior tibial nerve stimulation | N=42, 53.8, 0% | Unsuccessful trial of void after intervention requiring catheter (prior to discharge) |  |
|  |  | 2. Sham posterior tibial nerve stimulation | N=42, 56.4, 0% |  |  |
| Li 2010^53^  China | Hemorrhoid and fistula surgery  (n=60) | 1. Moxibustion | N=20, 44.2, 30% | Indwelling catheter within 24 hrs of treatment |  |
|  |  | 2. Hot compress | N=20, 42.2, 25% |  |  |
|  |  | 3. Infra-red radiation | N=20, 44.8, 30% |  |  |

**Table S5** **Summary estimates for the effects of interventions on the incidence of postoperative urinary retention together with GRADE ratings for certainty of the evidence**

| **Intervention** | **№ of participants (studies)** | | **Certainty of the evidence (GRADE)** | | **OR (95% CI)** | **Anticipated absolute effects** | |  |
| --- | --- | --- | --- | --- | --- | --- | --- | --- |
|  |  |  |  |  |  | **Risk with control** | **Risk difference with intervention** | **Number needed to treat (95% CI)** |
| **Pharmacological prevention of PO-UR** | | | | | | | |  |
| Avoidance of morphine | 210 (3 RCTs) | | ⨁⨁⨁◯^a^ | | 0.14 (0.06 to 0.34) | 317 per 1,000 | 256 fewer per 1,000 (290 fewer to 181 fewer) | NNTB 4(NNTB 4 to NNTB 6) |
| Morphine replacement | 100 (2 RCTs) | | ⨁⨁⨁⨁ | | 0.02 (0.01 to 0.10) | 580 per 1,000 | 553 fewer per 1,000 (566 fewer to 459 fewer) | NNTB 2(NNTB 2 to NNTB 3) |
| μ-opioid antagonists | 164 (2 RCTs) | | ⨁⨁⨁◯^b^ | | 0.49 (0.24 to 1.01) | 354 per 1,000 | 142 fewer per 1,000 (238 fewer to 2 more) | NNTB 8(NNTB 5 to NNTH 500) |
| Morphine administration | 52 (1 RCT) | | ⨁◯◯◯^d,b^ | | 0.69 (0.22 to 2.20) | 438 per 1,000 | 88 fewer per 1,000 (291 fewer to 194 more) | NNTB 12(NNTB 4 to NNTH 6) |
| NSAIDs | 343 (2 RCTs) | | ⨁⨁⨁◯^a^ | | 0.25 (0.10 to 0.65) | 130 per 1,000 | 94 fewer per 1,000 (115 fewer to 41 fewer) | NNTB 11(NNTB 9 to NNTB 25) |
| Anaesthesia: unilateral vs bilateral | 280 (2 RCTs) | | ⨁◯◯◯^e,f^ | | 0.17 (0.01 to 5.27) | 172 per 1,000 | 138 fewer per 1,000 (170 fewer to 350 more) | NNTB 8(NNTB 6 to NNTH 3) |
| Anaesthesia: epidural vs spinal | 120 (1 RCT) | | ⨁⨁⨁⨁ | | 0.35 (0.12 to 0.99) | 367 per 1,000 | 198 fewer per 1,000 (302 fewer to 2 fewer) | NNTB 6(NNTB 4 to NNTB 500) |
| Anaesthesia: general vs epidural | 80 (1 RCT) | | ⨁◯◯◯^b,d^ | | 0.37 (0.07 to 2.02) | 125 per 1,000 | 75 fewer per 1,000 (115 fewer to 99 more) | NNTB 14(NNTB 9 to NNTH 11) |
| Anaesthesia: local vs epidural | 112 (1 RCT) | | ⨁◯◯◯^d,f^ | | 0.47 (0.04 to 5.37) | 36 per 1,000 | 19 fewer per 1,000 (35 fewer to 129 more) | NNTB 53(NNTB 29 to NNTH 8) |
| Anaesthesia: pudendal nerve block vs spinal | 163 (1 RCT) | | ⨁⨁⨁⨁ | | 0.04 (0.01 to 0.09) | 695 per 1,000 | 612 fewer per 1,000 (673 fewer to 525 fewer) | NNTB 2(NNTB 2 to NNTB 2) |
| Clonidine vs fentanyl | 40 (1 RCT) | | ⨁⨁⨁◯^b^ | | 0.82 (0.24 to 2.84) | 500 per 1,000 | 49 fewer per 1,000 (306 fewer to 240 more) | NNTB 21(NNTB 4 to NNTH 5) |
| α-blockers | 1283 (12 RCTs) | | ⨁⨁⨁⨁ | | 0.24 (0.14 to 0.41) | 341 per 1,000 | 206 fewer per 1,000 (243 fewer to 166 fewer) | NNTB 5(NNTB 5 to NNTB 7) |
| α-blockers: Tamsulosin vs Alfuzosin | 120 (1 RCT) | | ⨁⨁⨁◯^b^ | | 0.74 (0.16 to 3.44) | 67 per 1,000 | 16 fewer per 1,000 (55 fewer to 131 more) | NNTB 63(NNTB 19 to NNTH 8) |
| Cholinergic drugs | 262 (3 RCTs) | | ⨁⨁◯◯^b,c^ | | 0.42 (0.12 to 1.46) | 318 per 1,000 | 154 fewer per 1,000 (265 fewer to 87 more) | NNTB 7(NNTB 4 to NNTH 12) |
| Antispasmodic | 201 (1 RCT) | | ⨁⨁⨁⨁ | | 0.29 (0.11 to 0.78) | 178 per 1,000 | 119 fewer per 1,000 (155 fewer to 34 fewer) | NNTB 9(NNTB 7 to NNTB 30) |
| Benzodiazepine | 90 (1 RCT) | | ⨁⨁◯◯^f^ | | 0.45 (0.04 to 5.10) | 47 per 1,000 | 25 fewer per 1,000 (45 fewer to 153 more) | NNTB 40(NNTB 23 to NNTH 7) |
| **Non-pharmacologic prevention** | | | | | | | |  |
| Mobilisation | 305 (2 RCTs) | | ⨁⨁⨁⨁ | | 0.35 (0.21 to 0.57) | 461 per 1,000 | 231 fewer per 1,000 (309 fewer to 133 fewer) | NNTB 5(NNTB 4 to NNTB 8) |
| IV Fluids | 193 (2 RCTs) | | ⨁⨁⨁◯^b^ | | 0.59 (0.26 to 1.34) | 447 per 1,000 | 124 fewer per 1,000 (273 fewer to 73 more) | NNTB 9(NNTB 4 to NNTH 14) |
| Oral Fluids | 496 (1 RCT) | | ⨁⨁◯◯^d^ | | 0.21 (0.09 to 0.45) | 149 per 1,000 | 114 fewer per 1,000 (134 fewer to 76 fewer) | NNTB 9(NNTB 8 to NNTB 14) |
| Pre-operative ultrasound monitoring | 281 (1 RCT) | | ⨁⨁◯◯^d^ | | 0.55 (0.31 to 0.98) | 271 per 1,000 | 101 fewer per 1,000 (168 fewer to 4 fewer) | NNTB 10(NNTB 6 to NNTB 250) |
| Electroacupuncture | 61 (1 RCT) | | ⨁⨁◯◯^f^ | | 0.18 (0.01 to 3.93) | 67 per 1,000 | 54 fewer per 1,000 (66 fewer to 153 more) | NNTB 19(NNTB 16 to NNTH 7) |
| External bladder stimulator | 43 (1 RCT) | | ⨁⨁⨁◯^b^ | | 1.38 (0.40 to 4.80) | 333 per 1,000 | 75 more per 1,000 (167 fewer to 373 more) | NNTH 14(NNTB 6 to NNTH 3) |
| Post-operative anal packing | 119 (1 RCT) | | ⨁⨁⨁◯ ^b^ | | 0.81 (0.35 to 1.92) | 246 per 1,000 | 37 fewer per 1,000 (143 fewer to 139 more) | NNTB 28(NNTB 7 to NNTH 8) |
| Straight catheterisation in recovery room | 54 (1 RCT) | | ⨁◯◯◯^a,b^ | | 1.40 (0.46 to 4.22) | 565 per 1,000 | 80 more per 1,000 (191 fewer to 281 more) | NNTH 13(NNTB 6 to NNTH 4) |
| Treatment of PO-UR |  | |  |  | |  |  |  |
| α-blockers | | 282 (2 RCTs) | ⨁◯◯◯^a,e,b^ | | 0.35 (0.08 to 1.44) | 520 per 1,000 | 245 fewer per 1,000 (440 fewer to 89 more) | NNTB 5(NNTB 3 to NNTH 12) |
| α-blocker vs carbachol +/- diazepam | | 261 (2 RCTs) | ⨁◯◯◯^a,e,b^ | | 0.49 (0.10 to 2.33) | 433 per 1,000 | 161 fewer per 1,000 (362 fewer to 207 more) | NNTB 7(NNTB 3 to NNTH 5) |
| Cholinergics +/- benzopiazepines | | 325 (4 RCTs) | ⨁◯◯◯^a,e^ | | not pooled | not pooled | not pooled |  |
| Warm gauze or hotpack | | 126 (2 RCTs) | ⨁⨁⨁⨁ | | 0.04 (0.01 to 0.14) | 929 per 1,000 | 586 fewer per 1,000 (814 fewer to 283 fewer) | NNTB 2(NNTB 2 to NNTB 4) |
| Warm gauze vs hotpack | | 84 (1 RCT) | ⨁⨁⨁◯ | | 0.59 (0.24 to 1.46) | 405 per 1,000 | 118 fewer per 1,000 (264 fewer to 93 more) | NNTB 9(NNTB 4 to NNTH 11) |
| Caffeine | | 30 (1 RCT) | ⨁⨁◯◯^f^ | | 0.12 (0.01 to 1.20) | 357 per 1,000 | 295 fewer per 1,000 (352 fewer to 43 more) | NNTB 4(NNTB 3 to NNTH 24) |
| Posterior tibial nerve stimulation | | 84 (1 RCT) | ⨁⨁⨁◯^b^ | | 0.45 (0.18 to 1.09) | 690 per 1,000 | 190 fewer per 1,000 (404 fewer to 18 more) | NNTB 6(NNTB 3 to NNTH 56) |
| Infrared radiation vs hot compress | | 40 (1 RCT) | ⨁⨁◯◯^b,g^ | | 0.82 (0.24 to 2.84) | 500 per 1,000 | 49 fewer per 1,000 (306 fewer to 240 more) | NNTB 21(NNTB 4 to NNTH 5) |
| Moxibustion vs hot compress | | 40 (1 RCT) | ⨁⨁⨁◯^g^ | | 0.11 (0.02 to 0.61) | 500 per 1,000 | 401 fewer per 1,000 (480 fewer to 121 fewer) | NNTB 3(NNTB 3 to NNTB 9) |
| Moxibustion vs infrared radiation | | 40 (1 RCT) | ⨁⨁⨁◯^g^ | | 0.14 (0.02 to 0.75) | 450 per 1,000 | 347 fewer per 1,000 (434 fewer to 70 fewer) | NNTB 3(NNTB 3 to NNTB 15) |
| **CI:** Confidence interval; **OR:** Odds ratio; a: At least 50% of trials at high risk of bias; b. CI includes 1; c. Moderate inconsistency; d. All trials at high risk of bias; e. substantial inconsistency; f. CI very wide and includes 1; g. publication bias detected  NNTB (Number needed to treat for one less case of PO-UR); NNTH (number needed to treat for one additional case of PO-UR) | | | | | | | | |
| **GRADE Working Group grades of evidence** ⨁⨁⨁⨁ **High certainty:** We are very confident that the true effect lies close to that of the estimate of the effect ⨁⨁⨁◯**Moderate certainty:** We are moderately confident in the effect estimate: The true effect is likely to be close to the estimate of the effect, but there is a possibility that it is substantially different ⨁⨁◯◯**Low certainty:** Our confidence in the effect estimate is limited: The true effect may be substantially different from the estimate of the effect ⨁◯◯◯**Very low certainty:** We have very little confidence in the effect estimate: The true effect is likely to be substantially different from the estimate of effect | | | | | | | |  |

# **Table S6 Secondary outcomes**

**Pharmacological Interventions**

**UTI**

|  | **Study** | **Intervention** | **Comparator** | **Population** | **Intervention**  **Events/participants** | **Comparator**  **Events/participants** | **OR (95% CI)** |
| --- | --- | --- | --- | --- | --- | --- | --- |
| **Morphine avoidance** | |  |  |  |  |  |  |
|  | Evron 1984 ^30^ | Epidural Bupivacaine | Epidural Bupivacaine & morphine | Caesarean | 1//40 | 4/40 | 0.23 (0.02 to 2.16) |
| **Anaesthesia** | | | |  |  |  |  |
|  | Evron 1984 ^30^ | General anaesthesia | Epidural Bupivacaine | Caesarean | 1//40 | 1/40 | 1.00 (0.06 to 16.56) |
| **α-blocker** | | |  |  |  |  |  |
|  | Lose 1985 ^55^ | Phenoxybenzamine | Placebo | Uterovaginal prolapse | 5/20 | 9/21 | 0.44 (0.12 to 1.68) |
|  | Evron 1984 ^30^ | Epidural Bupivacaine, morphine, phenoxybenzamine | Epidural Bupivacaine, morphine | Caesarean | 0/30 | 4/40 | 0.13 (0.01 to 2.57) |
|  | Petersen 1991 ^59^ | Prazosin hydrochloride | No treatment | Hip / Knee arthroplasty | 3/28 | 3/32 | 1.16 (0.21 to 6.27) |

**Pain**

|  | **Study** | **Intervention** | **Comparator** | **Population** | **Measure** | **Intervention*** | **Comparator*** | | **Effect estimate (95% CI)** | | |
| --- | --- | --- | --- | --- | --- | --- | --- | --- | --- | --- | --- |
| **Morphine replacement** | |  |  |  |  | |  |  | |  |  |
|  | Kim 2006 ^45^ | Sufentanil in ropivacaine | Morphine in ropivacaine | Gastrectomy | VAS coughing | 20.3(8.1; 30) | 19(7.6; 30) | | WMD 1.30 (-2.67 to 5.27) | | |
|  | Kim 2006 ^45^ | Sufentanil in ropivacaine | Morphine in ropivacaine | Gastrectomy | VAS resting | 17.3(7.8; 30) | 16.7(7.6; 30) | | WMD 0.60 (-3.30 to 4.50 | | |
| **α-blocker: Tamsulosin** | |  |  |  |  | |  |  | |  |  |
|  | Basheer 2017 ^19^ | Tamsulosin | Placebo | Spinal surgery |  | 3.9(2; 45) | 3.8(1.9; 46) | | WMD 0.10 (-0.70 to 0.90) | | |
|  | Cataldo 1991^26^ | Prazosin | Pleacebo | Anorectal surgery | Scale from 0 to 3 | No data available but reported no significant difference between treatment | | | | | |
| **Benzodiazepine** | | |  |  |  | |  |  | |  |  |
|  | Hershberger 2003 ^39^ | Lorazepam | Placebo saline | Gynaecology | VAS | 3.2(2; 48) | 2.2(1.9; 44) | | WMD 1.00 (0.20 to 1.80) | | |
| **Pudendal nerve block vs spinal anaesthesia** | | |  |  |  | |  |  | |  |  |
|  | Kim 2000^44^ | Pudendal nerve block | Spinal anaesthesia | Haemorrhoid surgery | # patients requiring analgesia | 16 /81 | 45 /82 | | OR 0.20 (0.10 to 0.41) | | |
|  |  |  |  |  | VAS | 2.7 (2.1; 81) | 5.2 (2.2; 82) | | WMD -2.50 (-3.16 to -1.84) | | |
| **NSAID suppository** | |  |  |  |  |  |  | |  | | |
|  | Placer Galan 2008^61^ | Diclofenac | No suppository | Haemorrhoidectomy | # patients requiring analgesia | 9 /73 | 20 /84 | | OR 0.45 (0.19 to 1.06) | | |

*Data presented as: Mean(SD; no. of patients) or no. of events/no. of participants

**Length of Hospital stay (hours)**

|  | **Study** | **Intervention** | **Comparator** | **Population** | **Intervention*** | **Comparator*** | **WMD (95% CI)** |
| --- | --- | --- | --- | --- | --- | --- | --- |
| **µ-opioid antagonist** | |  |  |  |  |  |  |
|  | Gallo 2008 ^31^ | Low-dose naloxone & morphine | Morphine | Orthopaedic | 74.4(31.2; 52) | 76.8(36; 45) | -2.40 (-15.91 to 11.11) |
| **Anaesthesia: Epidural vs Spinal** | | |  |  |  |  |  |
|  | Gao 2017 ^32^ | Bupivacaine epidural | Bupivacaine Subarachnoid | Lower limb surgery | 168(24; 30) | 151.92(57.6; 90) | 16.08 (1.40 to 30.76) |
| **Anaesthesia: Local vs Spinal** | | |  |  |  |  |  |
|  | Kau 2003 ^42^ | Epidural Lidocaine | Local Lidocaine with epinephrine | Haemorrhoidectomy | 3.75(1.55; 55) | 1.47(0.53; 57) | 2.28 (1.85 to 2.71) |
| **α-blocker: Phenoxybenzamine** | | |  |  |  |  |  |
|  | Lose 1985 ^55^ | Phenoxybenzamine | Placebo | Uterovaginal prolapse | 244.8(192-528; 20) | 252(168-672; 21) |  |
| **Benzodiazepine** | |  |  |  |  |  |  |
|  | Hershberger 2003 ^39^ | Lorazepam | Placebo saline | Gynaecology | 4.5(2.5; 48) | 3.7(2.2; 44) | 0.80 (-0.16 to 1.76) |

*Data presented as: Mean(SD; no. of participants); Mean(range; no. of participants).
**Non-Pharmacological Interventions**

**Pain**

| **Study** | | **Intervention** | **Comparator** | **Population** | **Intervention*** | **Comparator*** |
| --- | --- | --- | --- | --- | --- | --- |
| **Mobilisation** | |  |  |  |  |  |
|  | Hansen 2015 ^38^ | Mobilised in recovery room | No treatment | Disc herniation | 3(0-6; 67) | 2(0-7; 71) |
| **Bladder stimulator** | |  |  |  |  |  |
|  | Butwick 2003 ^23**^ | Bladder stimulator | Placebo | Knee Replacement | 1.5(0-8; 22) | 1(0-9; 21) |
|  | Butwick 2003 ^23***^ | Bladder stimulator | Placebo | Knee Replacement | 2.5(0-8; 22) | 2(0-8; 21) |

*Data presented as: Mean(range; no. of participants).
**8 hrs post theatre recovery discharge; ***3 hrs post theatre recovery discharge; Time outcomes reported as hours

**UTI**

| **Study** | | **Intervention** | **Comparator** | **Population** | **Intervention*** | **Comparator*** | **OR (95% CI)** |
| --- | --- | --- | --- | --- | --- | --- | --- |
| **Pre-operative ultrasound monitoring** | | |  |  |  |  |  |
|  | Joelsson-Alm 2012 ^41^ | Pre-op ultrasound monitoring | No pre-op scanning | Orthopaedic | 11/141 | 9/140 | 1.23 (0.49 to 3.07) |
| **Straight catheterisation in recovery room** | | |  |  |  |  |  |
|  | Hozack 1988 ^40^ | Catheterization in recovery room | No treatment | Hip / Knee arthroplasty | 5/31 | 2/23 | 2.02 (0.36 to 11.48) |

*Data presented as: no. of events/no. of participants

**Length of hospital stay**

| **Study** | | **Intervention** | **Comparator** | **Population** | **Intervention*** | **Comparator*** |
| --- | --- | --- | --- | --- | --- | --- |
| **Pre-operative ultrasound monitoring** | | |  |  |  |  |
|  | Joelsson-Alm 2012 ^41^ | Pre-op ultrasound monitoring | No pre-op scanning | Orthopaedic | 125(24-960; 141) | 125(24-480; 140) |

*Data presented as: Median(range; no. of participants)

## Appendix S2 Risk of bias by type of intervention

Risk of bias tables based on the assessment domains specified by the Cochrane Risk of Bias tool (ROB 2.0)^10, 11^:

- allocation bias (random sequence generation, allocation concealment and baseline imbalance)
- performance bias (departures from interventions, such as participant and study personnel blinding
- deviations from intended interventions and analysis in groups to which they were randomized)
- attrition bias (incomplete outcome data and robustness of results to missing data)
- detection bias (blinding of outcome assessors and likelihood of blinding to have influenced results)
- reporting bias (selective reporting of outcome domain being assessed)

Each study is rated as being at a “Low” or “High” risk, or as having “SC” (some concerns) of risk of bias for each individual domain. An overall risk of bias rating was assigned for each study based on the guidelines provided with ROB 2.0. Trials with interventions that fall into different categories appear in more than one table.

| **Pharmacological prevention** |  |  |  |  |  |  |
| --- | --- | --- | --- | --- | --- | --- |
| **Morphine avoidance** | **Allocation** | **Performance** | **Attrition** | **Detection** | **Reporting** | **Overall** |
| Tomaszewski 2014^66^ | **HIGH** | **HIGH** | **SC** | **LOW** | **LOW** | **HIGH** |
| El Dahab 2011^28^ | **LOW** | **LOW** | **LOW** | **LOW** | **LOW** | **LOW** |
| Evron 1984^30^, Evron 1984^29^* | **HIGH** | **SC** | **LOW** | **LOW** | **LOW** | **HIGH** |
| **Morphine replacement** |  |  |  |  |  |  |
| Kim 2006^45^ | **LOW** | **LOW** | **LOW** | **LOW** | **LOW** | **LOW** |
| Gentili 1996^34^ | **SC** | **SC** | **LOW** | **LOW** | **LOW** | **SC** |
| **μ-opioid antagonist** |  |  |  |  |  |  |
| Gallo 2008^31^ | **SC** | **SC** | **LOW** | **LOW** | **SC** | **SC** |
| Zand 2015^68^, Asadi 2012^17^ | **LOW** | **LOW** | **LOW** | **LOW** | **LOW** | **LOW** |
| **Morphine administration** |  |  |  |  |  |  |
| Petersen 1982^60^ | **HIGH** | **LOW** | **LOW** | **SC** | **LOW** | **HIGH** |

| **NSAID suppository** |  |  |  |  |  |  |
| --- | --- | --- | --- | --- | --- | --- |
| Khan 2015^43^ | **HIGH** | **SC** | **LOW** | **LOW** | **HIGH** | **HIGH** |
| Placer Galan 2008^61^ | **SC** | **LOW** | **LOW** | **LOW** | **LOW** | **SC** |
| **Anaesthesia: unilateral vs bilateral** |  |  |  |  |  |  |
| Ehrenberg 2010^27^ | **SC** | **SC** | **SC** | **SC** | **SC** | **SC** |
| Voelckel 2009^67^ | **LOW** | **LOW** | **LOW** | **LOW** | **LOW** | **LOW** |
| **Anaesthesia: epidural vs spinal** |  |  |  |  |  |  |
| Gao 2017^32^ | **LOW** | **LOW** | **LOW** | **LOW** | **LOW** | **LOW** |
| **Anaesthesia: general vs epidural** |  |  |  |  |  |  |
| Evron 1984^30^, Evron 1984^29^* | **HIGH** | **SC** | **LOW** | **LOW** | **LOW** | **HIGH** |
| **Anaesthesia: local vs epidural** |  |  |  |  |  |  |
| Kau 2003^42^ | **SC** | **HIGH** | **SC** | **LOW** | **LOW** | **HIGH** |
| **Anaesthesia: pudendal nerve block vs spinal** |  |  |  |  |  |  |
| Kim 2000^44^ | **SC** | **SC** | **LOW** | **SC** | **LOW** | **SC** |
| **Anaesthesia: clonidine vs fentanyl** |  |  |  |  |  |  |
| Kim 2001^49^ | **SC** | **SC** | **LOW** | **LOW** | **LOW** | **SC** |
| **α-blocker: Tamsulosin** |  |  |  |  |  |  |
| Bazzazi 2014^20^ | **SC** | **LOW** | **LOW** | **LOW** | **LOW** | **SC** |
| Basheer 2017^19^ | **LOW** | **LOW** | **SC** | **LOW** | **LOW** | **SC** |
| Akkoc 2016^16^ | **SC** | **LOW** | **LOW** | **SC** | **LOW** | **SC** |
| Mohammadi-Fallah 2012^58^ | **LOW** | **LOW** | **LOW** | **SC** | **SC** | **SC** |
| Madani 2014^57^, Madani 2011^56^ | **LOW** | **LOW** | **LOW** | **LOW** | **LOW** | **LOW** |
| **α-blocker: Phenoxybenzamine** |  |  |  |  |  |  |
| Evron 1984^30^, Evron 1984^29^* | **HIGH** | **SC** | **LOW** | **LOW** | **LOW** | **HIGH** |
| Lose 1985^55^ | **LOW** | **LOW** | **LOW** | **LOW** | **LOW** | **LOW** |
| Goldman 1988^35^ | **HIGH** | **SC** | **LOW** | **HIGH** | **LOW** | **HIGH** |
| **α-blocker: Prazosin** |  |  |  |  |  |  |
| Petersen 1991^59^ | **SC** | **SC** | **LOW** | **LOW** | **LOW** | **SC** |
| Cataldo 1991^26^ | **SC** | **SC** | **LOW** | **LOW** | **SC** | **SC** |
| Gonullu 1999^36^ | **SC** | **LOW** | **LOW** | **LOW** | **LOW** | **SC** |
| **α-blocker: Alfuzosin** |  |  |  |  |  |  |
| Akkoc 2016^16^ | **SC** | **LOW** | **LOW** | **SC** | **LOW** | **SC** |

| **α-blocker: Dibenzyline** |  |  |  |  |  |  |
| --- | --- | --- | --- | --- | --- | --- |
| Livne 1983^54^ | **HIGH** | **SC** | **LOW** | **LOW** | **LOW** | **HIGH** |
| **α-blocker: Tamsulosin vs Alfuzosin** |  |  |  |  |  |  |
| Akkoc 2016^16^ | **SC** | **LOW** | **LOW** | **SC** | **LOW** | **SC** |
| **Cholinergic** |  |  |  |  |  |  |
| Cameron 1966^24^ | **SC** | **LOW** | **LOW** | **LOW** | **LOW** | **SC** |
| Bowers 1987^21^ | **SC** | **SC** | **SC** | **LOW** | **SC** | **SC** |
| El Dahab 2011^28^ | **LOW** | **LOW** | **LOW** | **LOW** | **LOW** | **LOW** |
| **Anti-spasmodic** |  |  |  |  |  |  |
| Tomaszewski 2015^65^ | **SC** | **SC** | **LOW** | **SC** | **LOW** | **SC** |
| **Benzodiazepine** |  |  |  |  |  |  |
| Hershberger 2003^39^ | **LOW** | **LOW** | **LOW** | **LOW** | **LOW** | **LOW** |

| **Non-pharmacological prevention** |  |  |  |  |  |  |
| --- | --- | --- | --- | --- | --- | --- |
| **Mobilisation** | **Allocation** | **Performance** | **Attrition** | **Detection** | **Reporting** | **Overall** |
| Kim 1999^46^, Kim 1999^48^ | **SC** | **SC** | **LOW** | **LOW** | **LOW** | **SC** |
| Hansen 2015^38^ | **LOW** | **LOW** | **LOW** | **LOW** | **LOW** | **LOW** |
| **IV Fluids** |  |  |  |  |  |  |
| Lee 1999^52^ | **SC** | **SC** | **LOW** | **LOW** | **LOW** | **SC** |
| Kozol 1992^50^ | **SC** | **LOW** | **LOW** | **LOW** | **LOW** | **SC** |
| **Oral Fluids** |  |  |  |  |  |  |
| Bailey 1976^18^ | **HIGH** | **SC** | **LOW** | **LOW** | **SC** | **HIGH** |
| **Pre-operative ultrasound monitoring** |  |  |  |  |  |  |
| Joelsson-Alm 2012^41^ | **HIGH** | **LOW** | **LOW** | **LOW** | **LOW** | **HIGH** |
| **Electroacupuncture** |  |  |  |  |  |  |
| Gao 2014^33^ | **SC** | **SC** | **LOW** | **SC** | **LOW** | **SC** |
| **Bladder stimulator** |  |  |  |  |  |  |
| Butwick 2003^23^ | **LOW** | **LOW** | **LOW** | **LOW** | **LOW** | **LOW** |
| **Post-op anal packing** |  |  |  |  |  |  |
| Kim 2000^44^ | **SC** | **SC** | **LOW** | **LOW** | **LOW** | **SC** |
| **Straight catheterization in recovery room** |  |  |  |  |  |  |
| Hozack 1988^40^, Carpiniello 1988^25^ | **HIGH** | **LOW** | **HIGH** | **LOW** | **LOW** | **HIGH** |

| **Pharmacological treatment** |  |  |  |  |  |  |
| --- | --- | --- | --- | --- | --- | --- |
| **α-blocker** | **Allocation** | **Performance** | **Attrition** | **Detection** | **Reporting** | **Overall** |
| Tammela 1986^64^ | **LOW** | **SC** | **LOW** | **LOW** | **LOW** | **SC** |
| Burger 1997^22^ | **HIGH** | **LOW** | **LOW** | **LOW** | **LOW** | **HIGH** |
| **α-blocker vs carbachol +/- diazepam** |  |  |  |  |  |  |
| Burger 1997^22^ | **HIGH** | **LOW** | **LOW** | **LOW** | **LOW** | **HIGH** |
| Tammela 1986^64^ | **LOW** | **SC** | **LOW** | **LOW** | **LOW** | **SC** |
| **Cholinergic +/- benzodiazepines** |  |  |  |  |  |  |
| Gottesman 1989^37^ | **SC** | **LOW** | **LOW** | **LOW** | **LOW** | **SC** |
| Tammela 1986^64^ | **LOW** | **SC** | **LOW** | **LOW** | **LOW** | **SC** |
| Burger 1997^22^ | **HIGH** | **LOW** | **LOW** | **LOW** | **LOW** | **HIGH** |
| **Non-pharmacological treatment** |  |  |  |  |  |  |
| **Warm gauze or Hot pack** | **Allocation** | **Performance** | **Attrition** | **Detection** | **Reporting** | **Overall** |
| Afazel 2014^15^ | **SC** | **LOW** | **LOW** | **LOW** | **SC** | **SC** |
| **Caffeine** |  |  |  |  |  |  |
| Leach 2013^51^ | **SC** | **LOW** | **LOW** | **LOW** | **LOW** | **SC** |
| **Posterior tibial nerve stimulation** |  |  |  |  |  |  |
| Rardin 2011^63^ , Rardin 2006^62^ | **SC** | **SC** | **LOW** | **LOW** | **LOW** | **SC** |
| **Infrared radiation vs hot compress** |  |  |  |  |  |  |
| Li 2010^53^ | **SC** | **SC** | **LOW** | **LOW** | **LOW** | **SC** |
| **Moxibustion vs hot compress** |  |  |  |  |  |  |
| Li 2010^53^ | **SC** | **SC** | **LOW** | **LOW** | **LOW** | **SC** |
| **Moxibustion vs infrared radiation** |  |  |  |  |  |  |
| Li 2010^53^ | **SC** | **SC** | **LOW** | **LOW** | **LOW** | **SC** |
